# Editorial Note: The Pharmacological Chaperone AT2220 Increases Recombinant Human Acid α-Glucosidase Uptake and Glycogen Reduction in a Mouse Model of Pompe Disease

**DOI:** 10.1371/journal.pone.0334975

**Published:** 2025-10-22

**Authors:** The PLOS One Editors

After publication of this article [1], concerns were raised about the interpretation of Fig1B. Specifically, the fourth sentence of the Results sub-section titled AT2220 Stabilized rhGAA, Preventing Denaturation and Loss of Activity, reports a loss of GAA *activity* with a half-life of 3–4 hours in Fig 1B. However, upon editorial assessment the *PLOS One* Editors consider the data in Fig 1B to appear to be consistent with a GAA half-life of 13–14 hours.

Additionally, it is noted that the primary data underlying results in this article [1] are not included with the published article.

The authors did not respond to the journal’s communications.

The *PLOS One* Editors issue this Editorial Note to inform readers of the above discrepancy, which does not appear to affect the article’s main results and conclusions.

## References

[1] Khanna R, Flanagan JJ, Feng J, Soska R, Frascella M, Pellegrino LJ, et al. The pharmacological chaperone AT2220 increases recombinant human acid α-glucosidase uptake and glycogen reduction in a mouse model of Pompe disease. PLoS One. 2012;7(7):e40776. doi: 10.1371/journal.pone.0040776 22815812 PMC3399870

